# Butorphanol with oxygen insufflation corrects etorphine-induced hypoxaemia in chemically immobilized white rhinoceros (*Ceratotherium simum*)

**DOI:** 10.1186/s12917-014-0253-0

**Published:** 2014-10-15

**Authors:** Anna Haw, Markus Hofmeyr, Andrea Fuller, Peter Buss, Michele Miller, Gregory Fleming, Leith Meyer

**Affiliations:** Brain Function Research Group, School of Physiology, Faculty of Health Sciences, University of the Witwatersrand, Johannesburg, South Africa; Department of Paraclinical Sciences, Faculty of Veterinary Science, University of Pretoria, Pretoria, South Africa; Veterinary Wildlife Services, South African National Parks, Kruger National Park, Skukuza, South Africa; Division of Molecular Biology and Human Genetics, Faculty of Health Sciences, Stellenbosch University, Stellenbosch, South Africa; Disney’s Animal Programs and Environmental Initiatives, Lake Buena Vista, Florida USA; Centre for Veterinary Wildlife Studies, Department of Production Animal Studies, Faculty of Veterinary Science, University of Pretoria, Pretoria, South Africa

**Keywords:** Anaesthesia, Hypoxia, Hypercapnia, Acidaemia, Blood gases, Opioids, Partial-opioid antagonist

## Abstract

**Background:**

Opioid-induced immobilization is associated with severe respiratory depression in the white rhinoceros. We evaluated the efficacy of butorphanol and oxygen insufflation in alleviating opioid-induced respiratory depression in eight boma-managed rhinoceros.

**Results:**

Chemical immobilization with etorphine, azaperone and hyaluronidase, as per standard procedure for the white rhinoceros, caused severe respiratory depression with hypoxaemia (P_a_O_2_ = 27 ± 7 mmHg [mean ± SD]), hypercapnia (P_a_CO_2_ = 82 ± 6 mmHg) and acidosis (pH =7.26 ± 0.02) in the control trial at 5 min. Compared to pre-intervention values, butorphanol administration (without oxygen) improved the P_a_O_2_ (60 ± 3 mmHg, *F*_(3,21)_ =151.9, *p* <0.001), P_a_CO_2_ (67 ± 4 mmHg, *F*_(3,21)_ =22.57, *p* <0.001) and pH (7.31 ± 0.06, *F*_(3,21)_ =27.60, *p* <0.001), while oxygen insufflation alone exacerbated the hypercapnia (123 ± 20 mmHg, *F*_(3,21)_ =50.13, p <0.001) and acidosis (7.12 ± 0.07, *F*_(3,21)_ =110.6, *p* <0.001). Surprisingly, butorphanol combined with oxygen fully corrected the opioid-induced hypoxaemia (P_a_O_2_ = 155 ± 53 mmHg) and reduced the hypercapnia over the whole immobilization period (*p* <0.05, areas under the curves) compared to the control trial. However, this intervention (butorphanol + oxygen) did not have any effect on the arterial pH.

**Conclusions:**

Oxygen insufflation combined with a single intravenous dose of butorphanol improved the immobilization quality of boma-managed white rhinoceros by correcting the opioid-induced hypoxaemia, but did not completely reverse all components of respiratory depression. The efficacy of this intervention in reducing respiratory depression in field-captured animals remains to be determined.

## Background

Potent opioids, such as etorphine hydrochloride, are used routinely for the chemical immobilization of white rhinoceros (*Ceratotherium simum*), an essential procedure for the management and conservation of this species. Whilst these potent mu-agonist opioids induce a rapid immobilization, respiratory depression characterized by hypoventilation, hypoxaemia, hypercapnia and acidaemia is common [1-6]. The opioid-induced respiratory depression also is compounded by rigidity of the animal’s thoracic musculature, which impairs respiratory excursions [4]. Respiratory depression is arguably the most significant complication in the immobilization and anaesthesia of white rhinoceros [1,7,8], with animals shown to have an arterial oxygen partial pressure of less than 40 mmHg [2].

In an effort to reduce the respiratory depression, attempts have been made to selectively antagonize the undesirable side-effects of pure-opioid agonists while preserving their potent analgesic, sedative and catatonic effects, using mixed opioid agonists-antagonists such as nalorphine [7] and butorphanol [5,9]. Nalorphine is no longer readily available, so butorphanol has become widely used in the field [8]. However, whether butorphanol effectively reduces respiratory depression is not clear from the few studies that have been done [5,9]. Oxygen administered intranasally [10] or via nasotracheal intubation [3] also has been used to increase blood oxygen levels in etorphine-immobilized white rhinoceros, but field practitioners have questioned its efficacy and safety (Buss, personal communication).

In this paper, we critically evaluate combinations of butorphanol and oxygen insufflation in a controlled boma (small enclosure used to temporarily hold wild animals) setting to determine their efficacy in alleviating opioid-induced respiratory depression in white rhinoceros.

## Methods

This project was approved by the Animal Use and Care Committee, South African National Parks (SANParks) as well as the Animal Ethics Screening Committee of the University of the Witwatersrand (clearance 2012/23/04).

### Study area and sample population

Eight sub-adult (3–5 year old) male white rhinoceros were captured within Kruger National Park (−24.98984 S, 31.59263 E, Alt. 317 m) and housed individually in holding facilities (bomas) according to SANParks’ standard operating procedures. Food intake (lucerne and hay), defaecation and general demeanour were assessed daily for each rhinoceros. The trial started after a one month adaptation period. Data collection occurred from August to December 2012 and all immobilizations took place between 05H20 and 10H50. Black globe temperature (thermal load) ranged from 10°C to 43°C and barometric pressure varied from 737.0 to 745.9 mmHg.

### Chemical immobilization and experimental design

At each trial, the rhinoceros was immobilized with a combination of 2–3 mg etorphine hydrochloride (M99®, Novartis, Kempton Park, South Africa, 9.8 mg/ml), 30–45 mg azaperone (Stressnil®, Janssen Pharmaceutical Ltd., Halfway House, South Africa, 40 mg/ml), and 2500 i.u. hyaluronidase (lyophilized hyalase, Kyron Laboratories, South Africa). Drug doses were determined by body mass (Buss, personal communication, Table 1). Immobilizing drugs were administered into the nuchal hump using a 3 ml plastic dart with a 60 mm needle, fired with a CO_2_-powered dart-gun (Dan-Inject, South Africa). Once safe to handle the rhinoceros was blindfolded and positioned in lateral recumbency (Time 0) with padding under the head to ensure the lower nostril remained patent. Trials were performed only if the animal became immobile within 15 minutes of darting. Recumbency position was altered between left and right body side at each immobilization.

**Table 1:** **Drug doses for white rhinoceros immobilization (Buss)**

**Weight (kg)**	**Etorphine (mg)**	**Azaperone (mg)**	**Butorphanol (mg)**	**Naltrexone (mg)**
500-750	1.5	22.5	22.5	30
750-1000	2.0	30.0	30.0	40
1000-1250	2.5	37.5	37.5	50
1250-1500	3.0	45.0	45.0	60
1500-1750	3.5	52.5	52.5	70
1750-2000	4.0	60.0	60.0	80

The experiment consisted of four trials in which the interventions butorphanol (15 mg per mg etorphine, Kyron Laboratories, Benrose, South Africa, 20 mg/ml), butorphanol (15 mg per mg etorphine) + oxygen insufflation (30 L/min), oxygen insufflation (30 L/min) on its own, and sterile water (control) were evaluated and compared as measures to support the respiratory physiology of the immobilized white rhinoceros. Each rhinoceros received each intervention for the four trials in a randomised order at two-week intervals. The interventions were administered 6 min after the rhinoceros became laterally recumbent. Clinical data and samples were collected at 5 min after animals became laterally recumbent (before the intervention) and every 5 minutes thereafter for a 20-minute immobilization period. In the two trials where butorphanol was not administered at 6 min (oxygen only and control interventions), butorphanol (15 mg per mg etorphine) was administered at 21 minutes to facilitate arousal and loading into a crate.

### Clinical monitoring and data collection

Respiratory rate was monitored by an observer counting thoracic and abdominal excursions and feeling for expired air at the nares. The level of immobilization was assessed by observing body and ear movements at 5, 10, 15 and 20 min. The level of immobilization score ranged from 1 (no immobilizing or sedative effect) to 6 (excessive immobilization depth with respiration <3 breaths/min). Level 3 indicated a safe standing sedation, while levels 4 and 5 indicated recumbent immobilization with or without ear movement, respectively.

Arterial blood samples for blood gas analysis were collected from the medial auricular artery catheterized with a 22G × 1” IV catheter (Nipro Safelet Cath, Nipro Corporation) and secured in place with superglue (SuperGlue, Loctite®). A 0.5 ml sample was collected anaerobically into 1 ml heparinised syringes at 5, 10, 15 and 20 minutes after the rhinoceros became laterally recumbent. A final arterial sample was taken in the standing sedated rhinoceros, once positioned in a crate for weighing, 23 to 33 minutes after initial lateral recumbency. Arterial pH, partial pressures of carbon dioxide (P_a_CO_2_), and oxygen (P_a_O_2_), and haemoglobin oxygen saturation (S_a_O_2_, data not shown) were measured immediately after blood sampling using a portable precalibrated blood gas analyzer with precalibrated blood gas cassettes (Roche OPTI CCA Analyzer + OPTI cassette B, Kat Medical, Johannesburg, South Africa). The blood gas analyzer could not measure S_a_O_2_ values below 60%. The catheter was maintained with a heparinised (Heparin 5000 i.u./ml; Fresenius, Port Elizabeth, South Africa) saline flush and 1–2 ml of blood was discarded before collection of each sample.

During the experiments in which oxygen was delivered, nasotracheal intubation was achieved using an equine stomach tube (9.5 mm od × 213 cm, Kyron Laboratories) as described by Bush and colleagues [3]. Tracheal intubation was verified by hearing the air pass through the tube during respiration. Oxygen was delivered at a constant flow rate of 30 L/min based on earlier reports [3,4] and a prior pilot study.

Twenty-one minutes into the recumbent period, the rhinoceros was stimulated to stand and was guided into a crate for weighing. The quality of arousal was assessed according to the amount of stimulation needed to get the animal into a standing position and ability to guide the animal into a crate using an interval scale from 1 to 5. One indicates that the animal gets up too quickly, is too awake and cannot be blindfolded. 2 and 3 indicate that the animal gets up and can be safely handled and walked, with minor (2) or moderate (3) stimulation. 4 indicates that excessive stimulation is needed to get the animal standing and walking, while 5 indicates that the animal struggles to get up even after excessive stimulation and is unable to walk.

Approximately 35 min after the animals became laterally recumbent, the effects of etorphine were reversed using naltrexone (50 mg/ml, Kyron Laboratories, South Africa) administered intravenously into an auricular vein at 20-times the etorphine dose. The animals were then released from the crate into their bomas. The quality of recovery was subjectively categorized as poor, average or good by an individual veterinarian.

All rhinoceros used in these trials were monitored using a standardized boma scoring system on a daily basis for changes in appetite, defaecation, and behaviour. In addition, haematological and biochemical analyses were performed at the time of each immobilization to assess any changes in health status.

### Data analysis

Statistical analyses were performed using GraphPad Prism version 4.00 for Windows (GraphPad Software, San Diego, California, USA). All results are expressed as means ± SD, and statistical differences with *p* <0.05 were considered significant. For P_a_O_2_, P_a_CO_2_, pH and respiratory rate, a repeated measures two-way analysis of variance (ANOVA) followed by Bonferroni post-tests was used to test for differences between responses to butorphanol, butorphanol + oxygen, oxygen, and sterile water (control) at 5, 10, 15 and 20 min and between time points for each intervention. To determine the integrated response over time, areas under the response curves to butorphanol, butorphanol + oxygen, oxygen and sterile water (control) were calculated for these same variables (P_a_O_2_, P_a_CO_2_, pH and respiratory rate) for the period during lateral recumbency. A one-way ANOVA followed by a Student-Newman-Keuls (SNK) post hoc test was used to test for differences between these areas. The measurements taken while the animals were standing in the crate were analysed separately with a one-way ANOVA followed by a SNK post hoc test to compare differences between the trials in the standing rhinoceros.

## Results

All rhinoceros were in good health and showed behaviour indicative of healthy boma-managed rhinoceros throughout the trial. Although, initially after being relocated to the boma, the animals lost weight, they gained body mass gradually over time (average gain of 16 kg during the final two-week interval). The initial weight loss, associated with a brief period of anorexia, normally occurs while the animals adapt to captivity. Results of serum biochemical and haematological analyses were considered within normal ranges for healthy white rhinoceros [11]; Miller, personal communication. Follow-up monitoring of other health parameters subsequent to the experiment indicated no change in health status.

### Immobilization

The administration of etorphine (0.002 mg/kg), azaperone (0.03 mg/kg) and hyaluronidase (2500 i.u) led to rapid immobilization of rhinoceros (5 ± 1 minutes) after darting. There was no difference in time to recumbency between the four different trials (control, butorphanol, butorphanol + oxygen and oxygen) (*F*_(3,28)_ =0.95, *p* =0.43). The level of immobilization of the rhinoceros also did not differ significantly between different trials. When recumbent, the rhinoceros were usually relaxed with some ear movements (median immobilization level 4); occasionally they would enter a deeper state of immobilization where they would be fully relaxed without any ear movement (level 5).

### Respiratory rate

**Figure 1:**
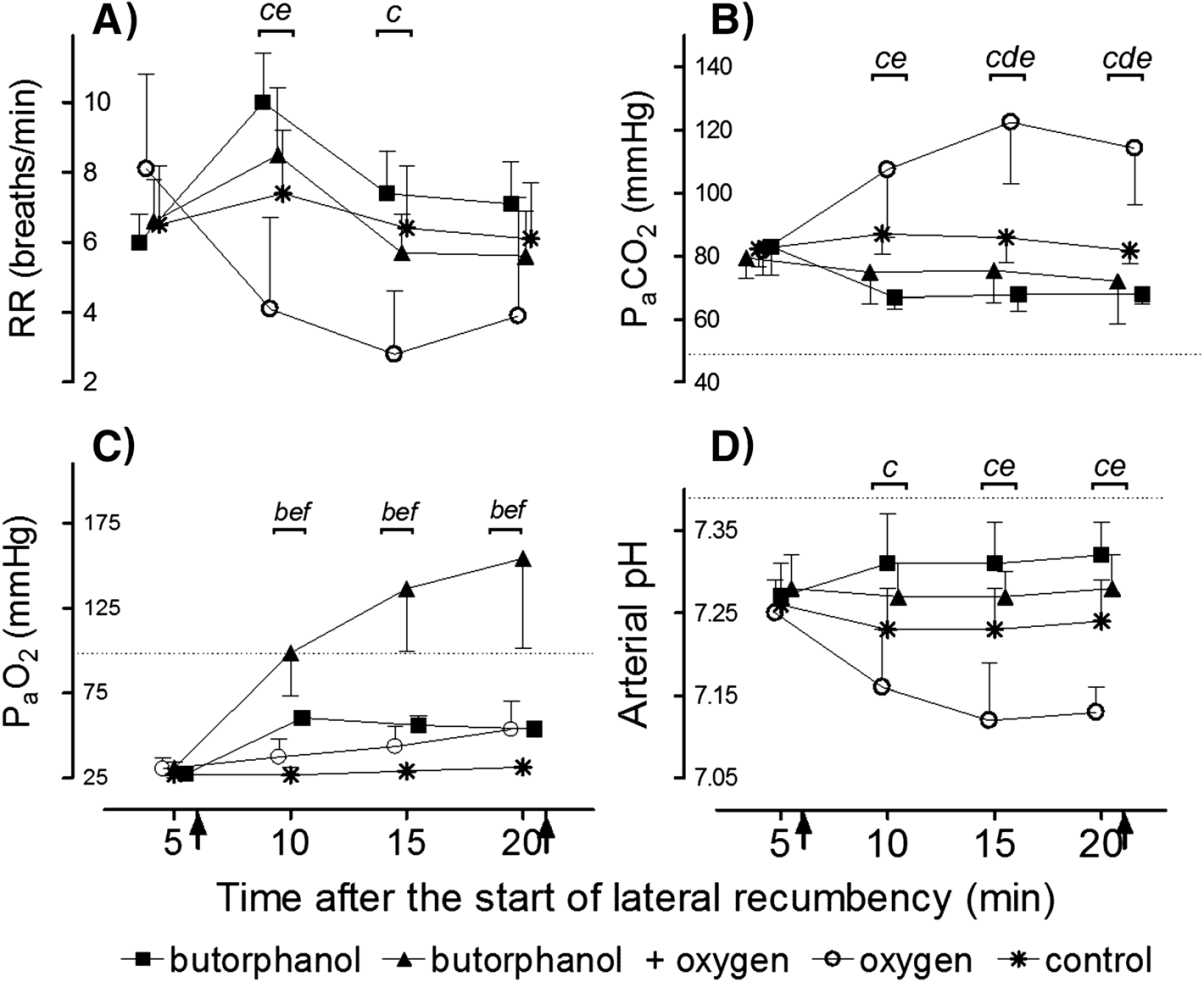
**Values are respiratory rate (A), partial pressure of arterial carbon dioxide (P**_**a**_ **CO**_**2**_ **) (B), partial pressure of arterial oxygen (P**_**a**_ **O**_**2**_ **) (C), and arterial pH (D) (means, SD,** ***n*** **=8) of rhinoceros given intravenous butorphanol, intravenous butorphanol + oxygen insufflation, oxygen insufflation and intravenous sterile water (control) 6 minutes after the rhinoceros became laterally recumbent.** Arrow at 6 minutes indicates time at which the intervention was given. Arrow at 21 minutes indicates the end of lateral recumbency. Dotted lines indicate average normal values in standing, unsedated white rhinoceros [12]. The brackets show when treatments differed from each other with letters indicating the treatment comparisons as follows: a = butorphanol vs. control; b = butorphanol vs. butorphanol + oxygen; c = butorphanol vs. oxygen; d = oxygen vs. control; e = oxygen vs. butorphanol + oxygen and f = butorphanol + oxygen vs. control.

**Figure 2:**
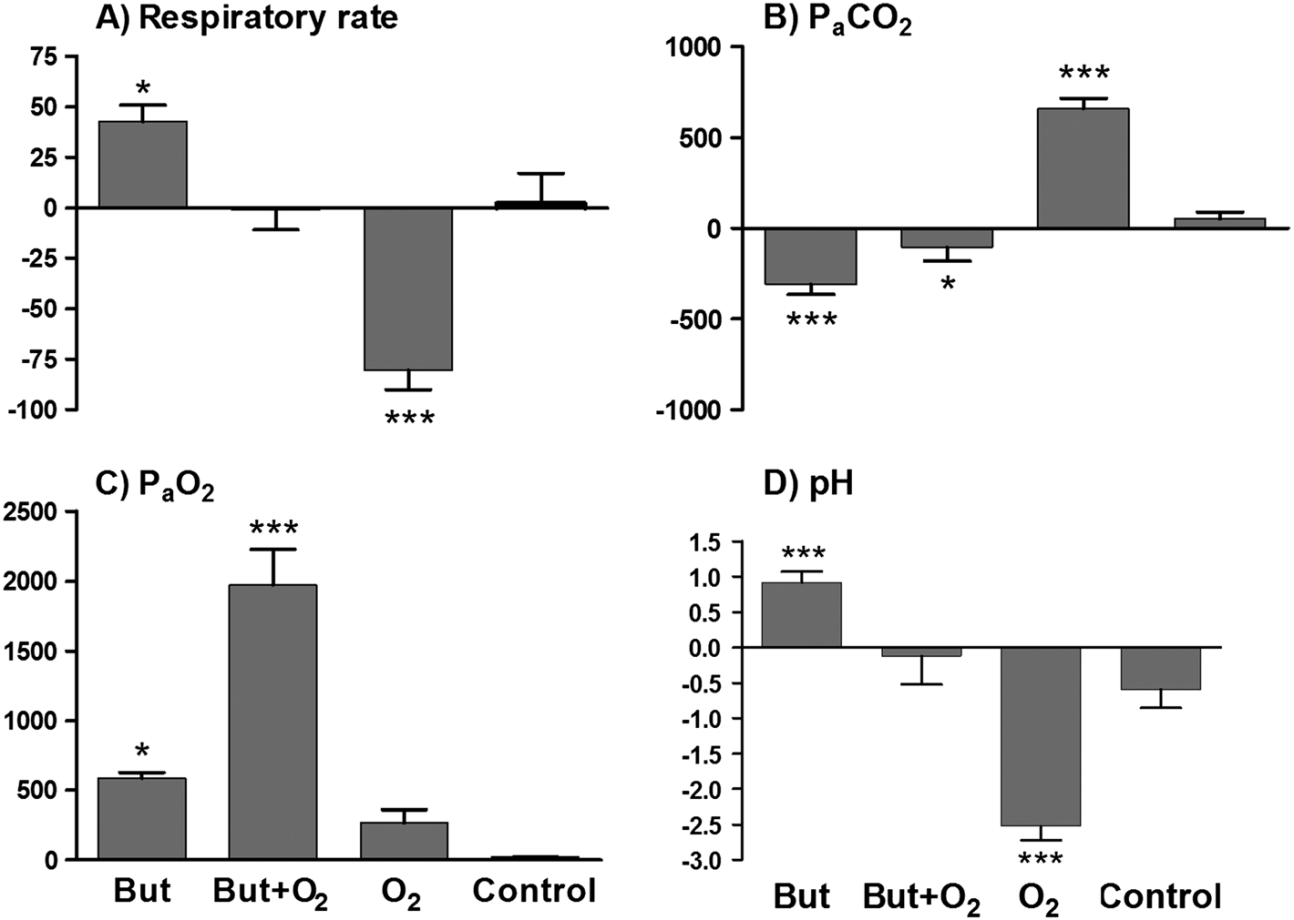
**Areas under the response curves.** Comparisons between the calculated values of the area under the response curves for respiratory rate **(A)**, partial pressure of arterial carbon dioxide (P_a_CO_2_) **(B)**, partial pressure of arterial oxygen (P_a_O_2_) **(C)** and arterial pH **(D)**. The asterix (*) indicates when the treatments butorphanol (But), butorphanol + oxygen (But + O_2_) or oxygen only (O_2_) differ significantly from the control, with the number of asterixes indicating the level of significance (*p <0.05, **p <0.01 and ***p <0.001).

Immobilization with etorphine, azaperone and hyaluronidase caused hypopnea (7 ± 2 breaths/min; normal mean [±SD] =19 [±0.6], breaths/min, [12]) which persisted throughout the immobilization period in animals that received the control (sterile water). There was no statistical difference (*F*_(3,28)_ =6.03, *p* >0.05) in mean respiratory rates between trials at t =5 min (butorphanol =6 ± 1; butorphanol + oxygen =7 ± 1; oxygen =8 ± 3 breaths/min) (Figure 1A). Although respiratory rate in the butorphanol trial significantly (*F*_(3,21)_ =48.30, *p* <0.001) increased following butorphanol administration to 10 breaths/min (SD 1) at t =10, this change was not different than mean respiratory rate for the control intervention (7 ± 2 breaths/min, t =10; *p* >0.05) (Figure 1A). However, when comparing the overall response over the immobilization period by comparing areas under the response curves (Figure 2A) respiratory rate did increase with butorphanol administration (*p* <0.05). Similarly, butorphanol combined with oxygen caused an initial increase in respiratory rate (9 ± 2 breaths per min at 10 min; *F*_(3,21)_ =8.97, *p* <0.01). However, this improvement was not sustained and the respiratory rate over the full 20-min immobilization period was not different from the control trial (Figure 2A). Oxygen insufflation as sole support caused a decrease in respiratory rate at 10, 15 and 20 min, compared to 5 min (*F*_(3,21)_ =37.62, *p* <0.001), and resulted in an overall lower respiratory rate than in the control trial (*p* <0.001, Figure 2A).

### Partial pressure of arterial carbon dioxide (P_a_CO_2_)

The decrease in respiratory rate induced by chemical immobilization resulted in severe hypoventilation, as indicated by the extreme hypercapnia (82 ± 6 mmHg, normal =49 [±0.9] mmHg [12]) at 5 min in the control trial, which did not change over time (F_*(3,21)*_ =2.68, *p* =0.073). The initial hypercapnia at 5 min was similar across rhinoceros in all trials (butorphanol =83 ± 9 mmHg; butorphanol + oxygen =79 ± 7 mmHg; oxygen =82 ± 8 mmHg) (Figure 1B). Butorphanol administration led to a decrease in P_a_CO_2_ from 5 to 10 min (67 ± 4 mmHg, *p* <0.001) which resulted in an overall improvement compared to the control (*p* <0.001, areas under the response curves, Figure 2B). The administration of butorphanol + oxygen did not result in an improvement in P_a_CO_2_ levels at 10, 15 or 20 min, compared to pre-intervention values (*F*_(3,21)_ =1.31, *p* >0.05, P_a_CO_2_ = 72 ± 13 mmHg at 20 min). However, over the full 20-min immobilization period, the P_a_CO_2_ was lower (*p* <0.05, areas under the response curves, Figure 2B) than that of the control. Oxygen insufflation as sole support had an immediate negative effect, increasing P_a_CO_2_ at 10 min to 106 ± 21 mmHg (*F*_(3,21)_ =50.13, *p* <0.001). At 15 min, P_a_CO_2_ peaked at 123 ± 20 mmHg, about 50% higher than the control value (*p* <0.001) at that time point (Figure 1B).

### Partial pressure of arterial oxygen (P_a_O_2_)

Chemical immobilization resulted in severe hypoxaemia (P_a_O_2_ = 27 ± 1 mmHg, normal =98.2 [±1.4] mmHg [12]) in the control trial at 5 min. This hypoxaemia at 5 min was similar across rhinoceros in all trials (butorphanol =28 ± 4 mmHg; butorphanol + oxygen =31 ± 6 mmHg; oxygen =30 ± 7 mmHg) (Figure 1C). Without any supportive treatment, oxygenation remained at extremely low levels (less than 35 mmHg) throughout the immobilization period. Butorphanol administration resulted in an immediate increase in P_a_O_2_ (60 ± 3 mmHg at 10 min; *F*_(3,21)_ =151.9, *p* <0.001), with better oxygenation compared to the control over the whole immobilization period (*p* <0.05, areas under the response curves, Figure 2C), although normoxia was not attained. Butorphanol + oxygen resulted in a marked and sustained increase in P_a_O_2_ (99 ± 26 mmHg at 10 min, *F*_(3,21)_ =33.36, *p* <0.001), which was greater than the control value at all time points (*p* <0.001) (Figure 1C). Indeed, butorphanol + oxygen increased P_a_O_2_ to levels above those normally seen in standing, unsedated white rhinoceros, and the increase was greater than butorphanol administration alone (butorphanol + oxygen =154 ± 53 mmHg; butorphanol =54 ± 4 mmHg at 20 min, *p* <0.001). Surprisingly, the overall improvement in P_a_O_2_ was greater than the combined effects of butorphanol alone and oxygen alone (Figure 2C). Administration of oxygen alone resulted in a delayed increase in P_a_O_2_ with an improvement seen only at 15 and 20 min (54 ± 17 mmHg, *F*_(3,21)_ =7.82, *p* <0.05) compared to pre-intervention values. However, oxygen insufflation did not result in an overall improvement in P_a_O_2_ compared to the control (*p* >0.05, areas under the response curves, Figure 2C).

### Arterial pH

Corresponding to the severe hypercapnia, animals that received the control intervention were acidaemic at 5 min (pH =7.26 ± 0.02, normal =7.39 ± 0.1 [12]) and their pH remained low throughout the immobilization period, with no difference between the pH at 5 min (7.26 ± 0.02) and 20 min (7.24 ± 0.05, *F*_(3,21)_ =4.64, *p* >0.05). The initial acidaemia at 5 min was similar across all treatment groups (butorphanol =7.27 ± 0.04; butorphanol + oxygen =7.28 ± 0.04; oxygen =7.25 ± 0.04) (Figure 1D). When butorphanol was administered, arterial pH improved initially, with pH at 10 min (7.31 ± 0.06) higher (*p* <0.001) than the pre-intervention pH. However, this improvement was not sustained and the rhinoceros remained acidaemic throughout the immobilization period. Overall, butorphanol led to an improvement in pH compared to the control (*p* <0.001, areas under the response curves, Figure 2D). Butorphanol + oxygen did not alter the pH at any time points following the intervention (*F*_(3,21)_ =0.55, *p* =0.66) and over the full 20-min immobilization period, the pH was not different from the control (Figure 2D). Oxygen alone caused a drop in pH from 7.25 ± 0.04 at 5 min to 7.13 ± 0.03 at 20 min (*F*_(3,21)_ =110.5, *p* <0.001), resulting in more severe acidaemia compared to the control and all other interventions (*p* <0.001, areas under the response curves, Figure 2D).

### Arousal

The median arousal score for rhinoceros after immobilization with butorphanol was 2, (animal gets up easily after minor stimulation, and when standing can be blindfolded, safely handled and walked without stimulation); while that for the control, oxygen and butorphanol + oxygen was 3 (animal gets up after moderate stimulation and when standing can be blindfolded, safely handled and walked with stimulation). Thus, rhinoceros that received only butorphanol at 6 min were aroused more easily at 26 min compared to rhinoceros that received the other treatments. Following the full reversal with naltrexone, all rhinoceros had a good or average recovery and returned to their bomas without complication.

### Arterial blood results from standing sedated rhinoceros

The last blood sample from the standing sedated rhinoceros while in the weighing crate, was taken, on average, 27 min (range 23–33 min) after the rhinoceros became recumbent. Following all the interventions, including the control, samples from the standing sedated rhinoceros indicated that the animals were still hypoxaemic (mean P_a_O_2_ values: control =69 ± 3 mmHg, butorphanol =58 ± 9 mmHg, butorphanol + oxygen =64 ± 8 mmHg, oxygen =87 ± 9 mmHg), hypercapnic (mean P_a_CO_2_ values: control =64 ± 4 mmHg, butorphanol =67 ± 3 mmHg, butorphanol + oxygen =67 ± 5 mmHg, oxygen =58 ± 4 mmHg) and acidaemic (mean pH values: control =7.33 ± 0.05, butorphanol =7.34 ± 0.03, butorphanol + oxygen =7.33 ± 0.02, oxygen =7.37 ± 0.05). However, the P_a_O_2_ values (87 ± 9 mmHg) from the animals that had received oxygen during immobilization, and then butorphanol one minute before walking, were higher than those in the butorphanol + oxygen (*p* <0.05) and butorphanol (*p* <0.01) groups.

## Discussion

We have shown that butorphanol administration in recently immobilized white rhinoceros attenuates the respiratory derangements that typically occur after etorphine-induced immobilization. However, the animals were still critically hypoxaemic (P_a_O_2_ = 53 ± 4 mmHg, normal P_a_O_2_ = 98.2 ± 1.4 mmHg [12]) 20 minutes into the immobilization period. The drug-induced hypoxaemia in white rhinoceros, however, could be corrected completely by administering intravenous butorphanol shortly after recumbency and supplying oxygen throughout the immobilization period via nasotracheal intubation. As far as we are aware, this is the first report of a protocol to completely reverse the severe hypoxaemia associated with etorphine-induced immobilization in white rhinoceros. However, the animals remained hypercapnic and acidaemic, illustrating that the immobilization-induced respiratory derangements were not entirely corrected. Surprisingly, we found that oxygen administered directly into the trachea at a flow rate of 30 L/min as sole support, decreased respiratory rate and exacerbated hypercapnia. Moreover, partial pressure of arterial oxygen was not statistically different from that of rhinoceros in the control group.

Arterial blood gas analysis is the gold standard for assessing the adequacy of oxygenation and ventilation. Although P_a_O_2_ and P_a_CO_2_ could be accurately determined, due to equipment logistics (uncuffed oxygen administration tubing), measurement of fractional inspired oxygen (F_i_O_2_) during oxygen interventions would have been inaccurate due to mixing of air with delivered oxygen. Therefore, the alveolar-arterial pressure gradient (P(A-a)O_2_) and other calculations could not be performed.

To our knowledge, this crossover trial is the first to critically evaluate, in a controlled environment, the respiratory physiology of the white rhinoceros during chemical immobilization. The effects of butorphanol [5,9] and oxygen insufflation [3] have been assessed previously during field immobilizations. However, as a result of uncontrolled variables (for example, dart placement, animal exertion) that occur in field trials, it is often difficult to establish definitive efficacy of a treatment, and comparing data from different field studies is thwart with error. Nevertheless, our study supports the common finding that etorphine immobilization in the white rhinoceros results in severe hypoxaemia together with hypercapnia and acidosis [1-3,5,7]. This hypoxaemia may be even more severe than previously reported, with rhinoceros sometimes having P_a_O_2_ values below 25 mmHg, less than a quarter of the normal value in an awake animal, five minutes into the immobilization period. Previously, the lowest reported P_a_O_2_ values were between 30 – 40 mmHg [2,3,5]. These negative effects of etorphine-induced immobilization on respiratory function are consistent with etorphine’s action as a potent mu-opioid receptor agonist.

Butorphanol is a mixed opioid agonist–antagonist, which has either mu-antagonist or partial mu-agonist and kappa-agonist activity [13]. Use in white rhinoceros immobilization is based on anecdotal evidence that butorphanol improves respiration by reversing the etorphine-induced mu-receptor activation [8]. However, Miller and colleagues [9] recently suggested that the beneficial effect of butorphanol may be related to a change in immobilization level rather than a direct antagonism of mu-receptors. Their conclusion was based on the persistence of hypoxaemia in rhinoceros given butorphanol during field immobilizations. In another study, immobilized white rhinoceros given nalorphine and diprenorphine, two other mixed opioid agonists-antagonists, also remained markedly hypoxaemic during immobilization [10]. Our finding that rhinoceros that received butorphanol 6 min after lateral recumbency were still hypoxaemic after 20 minutes of immobilization supports the idea that butorphanol does not reverse opioid-induced respiratory depression. However, in the rhinoceros in this study, ventilation did improve after butorphanol administration in comparison to the control, as indicated by lower P_a_CO_2_ and an improvement in P_a_O_2_. According to our ‘level of immobilization score’, butorphanol did not induce a lighter plane of immobilization compared to the control; however, we observed that some animals, although they remained completely immobile, were more responsive to gentle manipulation of the ear with this treatment. In future, additional criteria, such as response to a tactile stimulus, should be included in the ‘level of immobilization’ score. Butorphanol also appeared to reduce the etorphine-induced muscle tension and chest rigidity, an effect that may have accounted for improved minute ventilation.

In addition to butorphanol, another available treatment that has been advanced for improving oxygenation during rhinoceros immobilization is oxygen insufflation. In one study [3], the use of oxygen insufflation in field-immobilized white rhinoceros significantly increased arterial oxygen concentrations. Unexpectedly, our study contradicts these findings as we showed that oxygen as sole support did not improve P_a_O_2_ values compared to the control, and indeed had a detrimental effect on the respiratory status of immobilized white rhinoceros. This disparity in findings may be a result of intermittent use of a partial-opioid antagonist (nalorphine) and a respiratory stimulant (doxapram) during the field-based study [3], and other confounding variables associated with field trials.

Despite the variability between these studies, it is evident that hypoventilation, indicated by an increase in P_a_CO_2_, contributes to hypoxaemia in immobilized white rhinoceros. Another major contributing factor is likely to be ventilation-perfusion mismatch [1,5,10]. Previous studies have shown that P(A-a)O_2_ values in immobilized, recumbent white rhinoceros are often high [5]. Moreover, atelectasis has been found to occur after prolonged immobilization and recumbency in a white rhinoceros [1], and intrapulmonary shunts are a well-described complication of anaesthesia and recumbency in another large perissodactyl, the horse [14,15]. Together with intrapulmonary shunts, lateral recumbency in large quadrapeds leads to ventilation of under-perfused alveoli resulting in increased alveolar dead space. Indeed, in black rhinoceros, alveolar dead space was found to be greater in lateral, compared to sternally recumbent black rhinoceros [16]. Recumbency therefore most likely played an important role in the respiratory derangements of the rhinoceros in our study, but was not the primary cause of respiratory depression. We believe the primary cause was opioid-induced respiratory depression as the animal’s ventilatory response was inadequate to this severe hypoxaemia and hypercapnia.

The massive size of the rhinoceros undoubtedly contributes to atelectasis on the dependent side of the lung regions in laterally recumbent animals. However, absorption atelectasis, which occurs when less gas enters the alveolus than is removed by uptake by the blood, may be influenced by other factors such as gas composition [17]. The rate of absorption of gas from the alveoli into the blood increases with increasing F_i_O_2_ as oxygen is absorbed more quickly than nitrogen [17]. In humans with acute respiratory failure, mechanical ventilation with 100% oxygen causes an increase in intrapulmonary shunting primarily as a result of absorption atelectasis resulting from alveolar denitrogenation and pulmonary vasodilatation in unventilated lung areas [18-20]. Similarly, in horses under general anaesthesia where airway integrity is compromised by the weight of abdominal organs, increased intrapulmonary shunting occurs as a result of atelectasis when breathing high F_i_O_2_ (>0.8) compared to a lower F_i_O_2_ (0.21-0.3) [21,22]. It has also been demonstrated in horses receiving supplemental oxygen that lower minute ventilation will result in higher F_i_O_2_ [23]. Thus we propose that the rhinoceros receiving oxygen supplementation in our study had a high F_i_O_2_ together with narrowed or compressed airways as a result of pressure from abdominal organs and poor ventilation. In rhinoceros administered oxygen, gases in the alveoli were likely absorbed faster than they could be replaced and atelectasis ensued. If atelectasis is severe enough to cause shunt fractions in excess of 50%, oxygen supplementation will not be effective [10]. Therefore, we suggest that the rhinoceros receiving oxygen as the only intervention had significant intra-pulmonary shunt fractions, probably in the region of 50% or greater, as their P_a_O_2_ values were not significantly greater than the control.

When butorphanol was combined with oxygen insufflation, the partial pressure of arterial oxygen was substantially improved, and indeed entirely corrected with P_a_O_2_ reaching 154 ± 53 mmHg by the end of the recumbent period. This marked effect is difficult to explain given the negative effects of oxygen on its own, and the moderate improvements seen with butorphanol alone, but is most likely a result of the improved tidal volume and chest expansion induced by the butorphanol, which facilitated the high concentration of oxygen in the alveoli to dissolve into the blood without causing excessive atelectasis.

If atelectasis occurred in the rhinoceros that received oxygen only, it must have resolved quickly, as we found that, in standing rhinoceros, after the oxygen had been discontinued, P_a_O_2_ (87 ± 9 mmHg) was relatively close to normal levels. At least in one study investigating humans with acute respiratory failure [20], the loss of functional lung volume that occurred as a result of ventilation with 100% oxygen for 20 min, was immediately reversible in 19 of 20 patients. In our study, butorphanol was administered at the end of the recumbent period in the rhinoceros that received oxygen as sole support, so it is also likely that the butorphanol, together with stimulation and getting the animal’s into a standing position, led to improved ventilation and P_a_O_2_ values in these animals. On the other hand, rhinoceros that were given butorphanol at the beginning of the immobilization period, and were only stimulated to stand after 25 min, were still severely hypoxaemic (P_a_O_2_ = 58.4 ± 9.1 mmHg) in the standing position. This finding reveals that opioid-induced respiratory depression is a bigger complication than recumbency during white rhinoceros immobilization.

The partial pressure of arterial oxygen during oxygen administration may increase to a level at which hyperoxia occurs, thus affecting the ventilatory control system by reducing the output of peripheral and central chemoreceptors [24]. However, in our study, the rhinoceros that received inhalation oxygen as the sole support never became hyperoxic, but rather remained severely hypoxaemic. Normally, increasing levels of hypercapnia, as observed in these rhinoceros, should stimulate ventilation. However, full opioid agonists, such as etorphine, significantly reduce chemoreceptor sensitivity and therefore normal ventilatory control is inhibited during opioid-induced immobilization [13]. This inhibition of ventilatory control however, does not explain why the respiratory rate in rhinoceros receiving oxygen decreased from about 8 breaths per minute before oxygen administration to about 3 breaths per minute at 15 min when P_a_CO_2_ was at its highest (123 ± 12 mmHg) and P_a_O_2_ was only 44 ± 12 mmHg. Reasons for this further decrease in respiratory rate are not clear, but we hypothesize that it was directly related to the extreme hypercapnia resulting from a high shunt fraction, which caused a central depression of respiration [25].

This study has demonstrated that it is possible to fully correct the severe hypoxaemia that occurs in chemically-immobilized white rhinoceros. This is essential since inadequate tissue oxygenation may lead to an acute cessation of organ function resulting in morbidity, and even mortality [24]. However, although we managed to correct the hypoxaemia using butorphanol + oxygen, the intervention did not correct hypercapnia and acidaemia. Therefore, further investigations are needed to find treatments that fully support the respiratory physiology of immobilized white rhinoceros. Moreover, it is important to determine whether butorphanol combined with oxygen insufflation has the same positive effect on immobilization-induced hypoxaemia in field-immobilized white rhinoceros where increased exertion and other variables may alter individual responses.

## Conclusions

We compared the efficacy of butorphanol and oxygen insufflation, alone or in combination, for reversing opioid-induced respiratory depression in the chemically immobilized white rhinoceros. Butorphanol alone, although partly effective, did not prevent hypoxaemia and hypercapnia. Oxygen insufflation also did not reverse hypoxaemia or hypercapnia. Indeed, oxygen alone severely compromised the animal’s respiratory status, an effect we attribute to ventilation-perfusion mismatch and atelectasis.

Whilst the effects of butorphanol and oxygen administered alone were disappointing, oxygen insufflation combined with the intravenous administration of butorphanol at the beginning of the immobilization period completely reversed hypoxaemia. This supportive intervention therefore is likely to reduce, or even eliminate, the risk of hypoxic damage to vital organs and muscles, thereby improving the safety of white rhinoceros immobilization. Rhinoceros that received this treatment could be aroused with some stimulation, so that they could be guided into a loading crate, an important consideration when capturing large pachyderms. In the confined boma setting, where rhinoceros can be darted with ease and without excessive animal exertion, we recommend butorphanol and oxygen as the best available treatment to reverse hypoxaemia after etorphine administration. Whether the butorphanol and oxygen combination is equally effective in field-immobilized rhinoceros remains to be determined.

## Abbreviations

E_T_CO_2_: End-tidal carbon dioxide concentration
P_a_O_2_: Partial pressure of oxygen in the arterial blood
P_a_CO_2_: Partial pressure of carbon dioxide in the arterial blood
S_a_O_2_: Percentage of haemoglobin saturated with oxygen as measured in an arterial blood sample
SD: Standard deviation
SNK: Student-Newman-Keuls
